# Beamforming Design for Active-RIS-Aided Cell-Free Massive MIMO Networks Under Imperfect CSI

**DOI:** 10.3390/s26041286

**Published:** 2026-02-16

**Authors:** Qiang Ma, Hao Fang, Longxiang Yang

**Affiliations:** School of Communications and Information Engineering, Nanjing University of Posts and Telecommunications, Nanjing 210003, China; qiang_ma66@126.com (Q.M.); hao_f2022@163.com (H.F.)

**Keywords:** active RIS, 6G, cell-free massive MIMO, imperfect CSI, optimization

## Abstract

In the pursuit of an efficient 6G network that achieves an enhanced capacity with minimal power consumption, reconfigurable intelligent surfaces (RISs) and cell-free (CF) massive multiple-input-multiple-output (MIMO) networks emerge as two key technologies. This paper investigates an active-RIS-aided CF massive MIMO system under imperfect channel state information (CSI) and proposes a two-step optimization algorithm to address the max-min achievable rate problem. Given that the original problem is non-convex, we decompose it into two subproblems, which allows us to optimize the AP transmit beamforming and the RIS reflecting precoding, respectively, in an alternating manner. Simulation results demonstrate the superiority of the proposed scheme over existing benchmarks, achieving significant performance gains in active-RIS-aided CF massive MIMO systems.

## 1. Introduction

Rapid advancements of 6G wireless networks are driving extensive research efforts to explore innovative solutions to enhance energy sustainability and ultra-reliable coverage. Among these, cell-free massive multiple-input multiple-output (CF-mMIMO) has emerged as a key technology [1,2], where randomly distributed access points (APs) serve users, without conventional cell borders [3]. However, the performance of CF-mMIMO networks is dependent on favorable propagation conditions, which may be compromised in complex environments with severe blockages or deep fading.

In response, RIS have arisen as a key solution to manipulate the wireless channel [4,5,6]. Differing from the passive RIS, which only reflects incident signals without amplification, active RIS both adjusts the phase of reflected signals and amplifies them with gain. It is important to note that active RIS operates on a fundamentally different principle than conventional relays, as highlighted in [7]. Active RIS incorporates active components to mitigate the multiplicative fading effect, thereby significantly improving signal strength [8,9]. Integrating active RIS into CF-mMIMO networks presents a compelling approach to enhance both coverage and spectral efficiency, especially with high path loss or non-line-of-sight conditions.

However, the overall gains in active-RIS-aided CF-mMIMO systems critically depend on the accuracy of channel state information (CSI). In practical deployments, CSI is often imperfect owing to estimation inaccuracies, feedback latency, or hardware impairments [10,11]. Noise amplification in active-RIS-aided systems compounds the detrimental effects of imperfect CSI. This leads to increased channel estimation errors and, consequently, a degradation in overall system performance. Thus, it is imperative to develop beamforming optimization strategies that account for CSI uncertainties to guarantee reliable transmission.

### 1.1. Prior Works

The authors in [12] jointly optimized AP precoders and RIS phase shifts with the objective of maximizing the system-wide weighted sum-rate (WSR), considering imperfect CSI in passive RIS-aided CF networks. The problem of max-min fairness, specifically the maximization of the minimum achievable rate in a passive-RIS-aided CF network, was studied in [13]. In [14], the authors proposed a physical layer secrecy transmission scheme employing an active RIS with multiple antennas. Their work demonstrates the active RIS can effectively mitigate the “double fading” phenomenon and attain higher energy efficiency against the passive RIS. In [15], the authors investigated an active-RIS-aided cell-free network and proposed a joint beamforming and resource allocation algorithm aimed at maximizing the minimum user energy efficiency. The authors in [16] introduced a sub-connected architecture for active RIS, aiming to enhance power efficiency. In [17], the authors explored the potential of active RISs to facilitate secure communication. The authors in [18] investigated a two-timescale transmission strategy for CF-mMIMO assisted by RIS, accounting for imperfect channel. A linear minimum mean square error estimator was designed to acquire the aggregated instantaneous CSI. The authors in [19] introduced an analysis of achievable rates along with a phase-shift optimization algorithm that relies on statistical CSI for multi-cell systems assisted by RIS and MIMO technology. The authors in [20] investigated energy efficiency maximization through the optimization of transmit beamforming and multi-functional RIS coefficients, taking into account both statistical and bounded CSI error models. In [21], the authors introduced an optimal channel estimation protocol grounded in minimum mean squared error principles and introduced an algorithm for the maximization of the minimum SINR. 

### 1.2. Contributions

From the above discussion, prior researchers do not jointly consider the implications of imperfect CSI and active RIS in CF-mMIMO systems. Differing from existing articles, our study centers on integrating active RISs into a CF-mMIMO network under imperfect channel state information.

The primary contributions of this paper lie in the following aspects:

(1) Our work investigates the performance of active RIS-aided CF-mMIMO systems with imperfect CSI by accounting for channel estimation errors. To improve the max-min fairness, we formulate an optimization problem to jointly optimize the AP transmit beamforming and RIS reflecting precoding, aiming to maximize the minimum achievable rate.
(2) Given the challenge of the max-min problem, we divide it into two subproblems. Then, we use the second-order cone programming (SOCP) and semidefinite relax (SDR) method to solve the subproblems iteratively, acquiring the convergence solution of this problem.

### 1.3. Organization

This paper is presented as follows. Section 2 outlines the system and channel models and establishes an optimization problem focused on max-min achievable rate maximization. Section 3 proposes a two-step algorithm to address this problem. Section 4 presents and discusses simulation results that assess the algorithm. Finally, Section 5 draws the conclusions of this article. 

### 1.4. Notation 

Within this article, a bold uppercase letter **G** denotes a matrix, and column vectors are denoted by a bold lowercase letter **g**. The superscripts $({)}^{T}$ and $({)}^{H}$ are used to denote transpose and conjugate transpose, respectively. We use $I_{N}$ for the N×N identity matrix, tr(·) for the trace of the matrix and diag(·) for the diagonalization. The norm of the vector g is denoted by $\left\|g\right\|$. Finally, we denote a circularly symmetric complex Gaussian random column vector **x** with mean **a** and covariance matrix **B** by x~CN(a,B).

## 2. System Model

**Figure 1:**
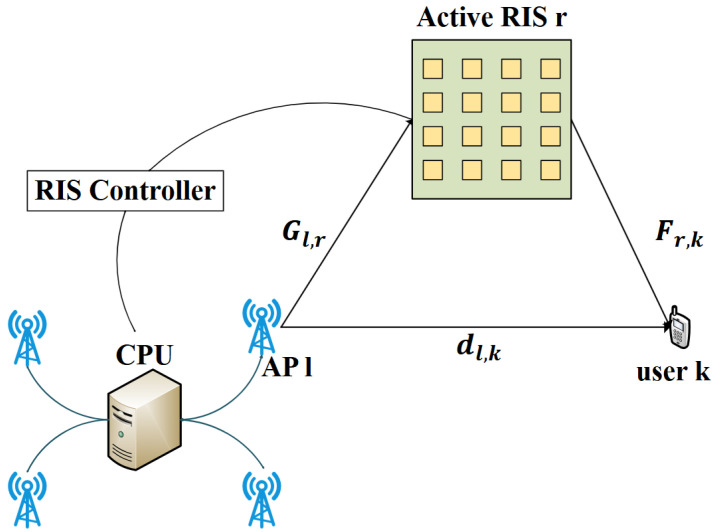
Schematic of the active RIS-aided CF-mMIMO system.

We investigate a CF-mMIMO network assisted by the active RIS, including L APs, R RISs and K single-antenna users. We assume each AP has an array of M antennas. Each RIS comprises N active reflecting elements. The AP and RIS are wirelessly connected to a CPU that coordinates them. Figure 1 depicts the specific system schematic.

### 2.1. Channel Models

The aggregated perfect channel from AP l to user k is formulated as:

(1) $$h_{l,k}^{H}=d_{l,k}^{H}+\sum_{r=1}^{R}F_{r,k}^{H}\Theta_{r}G_{l,r}$$

where $d_{l,k}^{H}\in C^{1\times M}$ denotes the direct downlink channel from the lth AP to the kth user. $\;F_{r,k}^{H}\in C^{1\times N}$ is the channel between the rth active RIS and the kth user. $G_{l,r}\in C^{N\times M}$ is the channel from the lth AP to the rth active RIS. $\Theta_{r}=diag(\beta_{1}e^{j\varphi_{r,1}},\beta_{2}e^{j\varphi_{r,2}},\cdots ,\beta_{N}e^{j\varphi_{r,N}})$ is the reflection coefficient matrix at the rth RIS. $\beta_{n}$ denotes the reflection amplitude, and $\varphi_{r,n}$ represents the phase shift in the nth element at the rth RIS, which is a continuous variable ranging from 0 and 2π.

The sensitivity of CSI leads to residual CSI errors, which further impair the overall performance. Moreover, faulty elements within an RIS can degrade the channel estimation accuracy for both direct and reflected links. The relationship of the perfect to imperfect CSI can be described as follows [22,23]:

(2) $${\overset{̑}{h}}_{l,k}^{H}=\sqrt{1-\xi_{k}}h_{l,k}^{H}+\sqrt{\xi_{k}}{\tilde{h}}_{l,k}^{H}$$

where ${\overset{̑}{h}}_{l,k}^{H}\in C^{1\times M}$ and $h_{l,k}^{H}\in C^{1\times M}$ represent the estimated and the correct channel given in (1), respectively. ${\tilde{h}}_{l,k}^{H}$ is residual CSI error with independent and identically distributed zero-mean and unit-variance complex Gaussian. Additionally, $\xi_{k}$ denotes the error factor of user k, ranging from 0 to 1, which reflects the accuracy of the CSI. This simplified model effectively captures the aggregate impact of imperfect CSI by assuming the estimation error is statistically independent of the true channel [24]. The scalar factor $\xi_{k}$ serves as a composite indicator of the overall channel estimation quality for user k, which typically degrades with lower pilot power, higher mobility, or increased interference. This model offers a tractable foundation for deriving closed-form performance expressions (e.g., the achievable rate), which is crucial for the subsequent beamforming design and analysis.

### 2.2. Transmitters and Receivers

The signal to be transmitted by the lth AP is

(3) $$x_{l}=\sum_{k=1}^{K}w_{lk}s_{k}$$

where $w_{l,k}\in C^{M\times 1}$ is the beamforming vector utilized by the lth AP. In addition, the transmit power of the lth AP must fulfill this constraint:

(4) $$\sum_{j=1}^{K}{\left\|w_{l,j}\right\|}^{2}\leq P_{l}\;\;\;\;\;\;\;\;\;\forall l\in L$$

where $P_{l}$ denotes the maximum allowable power at the lth AP. $s=[s_{1},\cdots ,s_{K}{]}^{T}\in C^{K\times 1}$ denotes the intended signals for the kth user, satisfying $E[{\left|s_{k}\right|}^{2}]=1$.

Let $y_{k}$ denote the signal received at the kth user, which is expressed as:

(5) $$y_{k}=\sqrt{1-\xi_{k}}h_{k}^{H}w_{k}s_{k}+\sum_{\begin{array}{c} j=1\\ j\neq k \end{array}}^{K}\sqrt{1-\xi_{k}}h_{k}^{H}w_{j}s_{j}+\sum_{j=1}^{K}\sqrt{\xi_{k}}{\tilde{h}}_{k}^{H}w_{j}s_{j}+\sum_{r=1}^{R}F_{r,k}^{H}\Theta_{r}z_{r}+n_{k}$$

where $h_{k}=[h_{1,k}^{T},\cdots ,h_{L,k}^{T}{]}^{T}\in C^{LM\times 1}$, $w_{k}=[w_{1,k}^{T},\cdots ,w_{L,k}^{T}{]}^{T}\in C^{LM\times 1}$, ${\tilde{h}}_{k}=[{\tilde{h}}_{l,1}^{T},\cdots ,{\tilde{h}}_{l,K}^{T}{]}^{T}\in C^{LM\times 1}$. $z_{r}\sim CN(0,\sigma^{2}I_{N})$ represents dynamic noise influenced by active RIS. In addition, $n_{k}$ is the additive complex white Gaussian noise, with zero mean and variance $\sigma_{0}^{2}$. 

Achievable rate for the kth user is $r_{k}=\log_{2}(1+\mathrm{SIN}R_{k})$. Specifically, the effective SINR for the kth user is given by:

(6) $$\mathrm{SIN}R_{k}=\frac{(1-\xi_{k}){\left|h_{k}^{H}w_{k}\right|}^{2}}{\sum_{\begin{array}{c} j=1\\ j\neq k \end{array}}^{K}(1-\varsigma_{k}){\left|h_{k}^{H}w_{j}\right|}^{2}+\sum_{j=1}^{K}\xi_{k}{\left|{\tilde{h}}_{k}^{H}w_{j}\right|}^{2}+{\left\|F_{k}^{H}\Theta \right\|}^{2}\sigma^{2}+\sigma_{0}^{2}}$$

where $\Theta =diag[\Theta_{1},\Theta_{2},\cdots ,\Theta_{R}]$, $F_{k}^{H}=[F_{1,k}^{H},F_{2,k}^{H},\cdots ,F_{R,k}^{H}]$.

### 2.3. Problem Formulation

We pose the optimization problem as follows:

(7a) $$P(1):\;\underset{W,\Theta ,\xi }{max}\;\underset{\forall k\in K}{min}r_{k}$$

(7b) $$st.\;\sum_{j=1}^{K}{\left\|w_{l,j}\right\|}^{2}\leq P_{l}\;\;\;\;\;\;\;\;\forall l\in L$$

(7c) $$\sum_{l=1}^{L}\sum_{j=1}^{K}{\left\|\Theta_{r}G_{l,r}w_{l,j}\right\|}^{2}+{\left\|\Theta_{r}\right\|}^{2}\sigma^{2}\leq P_{R}\;\;\;\;\;\;\;\;\forall r\in R$$

(7d) $$0\leq \xi_{k}\leq 1$$

where $W=[w_{1}^{T},w_{2}^{T},\cdots w_{K}^{T}{]}^{T}$ denotes the aggregate transmit beamforming matrix from all APs to all users. $P_{l}$ and $P_{R}$ represent transmit power limit per AP and RIS amplification power budget, respectively.

## 3. Proposed Alternating Optimization

To decouple the interdependent beamforming and reflection variables in P(1), this section decomposes the original problem into two tractable subproblems.

### 3.1. AP Transmit Beamforming Design

For any given Θ and $\xi_{k}$, We can transform P(1) in the following form:

(8a) $$P(2):\;\underset{W}{\max}\underset{\forall k\in K}{\min}R_{k}$$

(8b) $$st.\;\sum_{j=1}^{K}{\left\|w_{l,j}\right\|}^{2}\leq P_{l}\;\;\;\;\;\;\;\;\forall l\in L$$

(8c) $$\sum_{l=1}^{L}\sum_{j=1}^{K}{\left\|\Theta_{r}G_{l,r}w_{l,j}\right\|}^{2}+{\left\|\Theta_{r}\right\|}^{2}\sigma^{2}\leq P_{R}\;\;\;\;\;\;\;\;\forall r\in R$$

With the introduction of an auxiliary variable t, a reformulated version of P(2) is given by:

(9a) $$P(3):\;\underset{W}{\max}\;t$$

(9b) $$st.\;\sum_{j=1}^{K}{\left\|w_{l,j}\right\|}^{2}\leq P_{l}\;\;\;\;\;\;\;\;\forall l\in L$$

(9c) $$\sum_{l=1}^{L}\sum_{j=1}^{K}{\left\|\Theta_{r}G_{l,r}w_{l,j}\right\|}^{2}+{\left\|\Theta_{r}\right\|}^{2}\sigma^{2}\leq P_{R}\;\;\;\;\;\;\;\;\forall r\in R$$

(9d) $$(1-\xi_{k}){\left|h_{k}^{H}w_{k}\right|}^{2}\geq t(\sum_{\begin{array}{c} j=1\\ j\neq k \end{array}}^{K}(1-\varsigma_{k}){\left|h_{k}^{H}w_{j}\right|}^{2}+\sum_{j=1}^{K}\xi_{k}{\left|{\tilde{h}}_{k}^{H}w_{j}\right|}^{2}+{\left\|F_{k}^{H}\Theta \right\|}^{2}\sigma^{2}+\sigma_{0}^{2})$$

According to [25,26], we exploit the phase ambiguity to rotate the phase such that the inner product $h_{k}^{H}W_{k}$ is positive. Also, we define $A=diag[A_{1},A_{2},\cdots ,A_{L}]$ and $A_{i}=G_{ir}^{H}\Theta_{r}^{H}\Theta_{r}G_{ir}$. On this basis, P(3) can be rewritten as:

(10a) $$P(4):\;\underset{W}{\max}\;t$$

(10b) $$st.\;W^{H}CW\leq P_{l}\;\;\;\;\;\;\;\;\forall l\in L$$

(10c) $$W^{H}EW\leq P_{R}-{\left\|\Theta_{r}\right\|}^{2}\sigma^{2}\;\;\;\;\;\;\;\;\forall r\in R$$

(10d) $$\begin{array}{c} R(\sqrt{1-\xi_{k}}h_{k}^{H}w_{k})\geq \\ \sqrt{t}\left\|\sqrt{1-\xi_{k}}(h_{k}^{H}W_{k}),\sum_{j=1}^{K}\sqrt{\xi_{k}}({\tilde{h}}_{k}^{H}w_{j}),F_{k}^{H}\Theta \sigma ,\sigma_{0}\right\| \end{array}$$

where $E=I_{K}⊗A$, $W_{k}=[w_{1},w_{2},\cdots w_{k-1},w_{k+1},\cdots ,w_{K}]$, $C=I_{k}⊗(\xi_{b}^{H}\xi_{b})⊗I_{M}$. The vector $\xi_{b}\in R^{1\times L}$ represents the bth element is 1 and all other elements are 0.

The constraints of (10b), (10c) and (10d) belong to the second-order cone (SOC) type. Therefore, the bisection search is employed to solve P(4), in which each step involves solving a feasibility problem of SOCP [27]. The specific algorithm proposed is detailed in Algorithm 1.
**Algorithm 1:** Proposed Algorithm for Addressing P(4)**Input:** All channels $d_{l,k}^{H},G_{l,r},\;F_{r,k}^{H}$, the initial Θ and $\xi_{k}$. **Output:**
$W_{opt}$ 1: Initialize the minimum and maximum bounds $t_{\min}$ and $t_{\max}$ for the variable in the objective function mentioned in (10a). Choose a tolerance δ=0.1. 2: Set $t=(t_{\min}+t_{\max})/2$. Solve the SOCP problem using the CVX solver:                    find: W                           s.t. $W^{H}CW\leq P_{l}\;\;\;\;\;\;\;\;\forall l\in L$                                                             (11a)                                 $W^{H}EW\leq P_{R}-{\left\|\Theta_{r}\right\|}^{2}\sigma^{2}\;\;\;\;\;\;\;\;\forall r\in R$                                          (11b)                                 $\begin{array}{c} R(\sqrt{1-\xi_{k}}h_{k}^{H}w_{k})\geq \\ \sqrt{t}\left\|\sqrt{1-\xi_{k}}(h_{k}^{H}W_{k}),\sum_{j=1}^{K}\sqrt{\xi_{k}}({\tilde{h}}_{k}^{H}w_{j}),F_{k}^{H}\Theta \sigma ,\sigma_{0}\right\| \end{array}$                      (11c)
3: If the above problem is solved, then assign $t_{\min}=t$, otherwise, set $t_{\max}=t$. 4: Stop and output $W_{opt}$ when $t_{\min}-t_{\max}\leq \delta$. If not, proceed to Step 2.

The complexity of Algorithm 1 is primarily due to the solution of the SOCP, solvable via an interior-point method. As indicated in [28], the complexity required to solve such an SOCP problem is $O(\sqrt{r}(p^{3}+p^{2}\sum_{i=1}^{r}q_{i}+\sum_{i=1}^{r}q_{i}^{2}))$, where p, r, and $q_{i}$ are the count of variables, the number of SOCP constraints, and the size of the ith SOCP, respectively. Consequently, Algorithm 1 has a computational complexity of $O(Iter_{SOCP}\sqrt{L+K}(2L^{3}K^{3}+L^{2}K^{4}+K^{3}+LK^{2}))$, where $Iter_{SOCP}$ represent the iteration count required for Algorithm 1 to converge.

### 3.2. RIS Reflecting Precoding Design

For given W and $\xi_{k}$, P(1) is reformulated into:

(12a) $$P(5):\;\underset{\Theta_{r}}{\max}\;\underset{\forall k\in K}{\min}r_{k}$$

(12b) $$st.\;\sum_{l=1}^{L}\sum_{j=1}^{K}{\left|\Theta_{r}G_{lr}w_{lj}\right|}^{2}+{\left|\Theta_{r}\right|}^{2}\sigma^{2}\leq P_{R}\;\;\;\;\;\;\;\;\forall r\in R$$

With the introduction of an auxiliary variable $t\in R^{+}$, P(5) is reformulated as follows:

(13a) $$P(6):\;\underset{\Theta_{r}}{\max}\;t$$

(13b) $$st.\;\sum_{l=1}^{L}\sum_{j=1}^{K}{\left|\Theta_{r}G_{lr}w_{lj}\right|}^{2}+{\left|\Theta_{r}\right|}^{2}\sigma^{2}\leq P_{R}\;\;\;\;\;\;\;\;\forall r\in R$$

(13c) $$\frac{(1-\xi_{k}){\left|h_{k}^{H}w_{k}\right|}^{2}}{\sum_{\begin{array}{c} j=1\\ j\neq k \end{array}}^{K}(1-\varsigma_{k}){\left|h_{k}^{H}w_{j}\right|}^{2}+\sum_{j=1}^{K}\xi_{k}{\left|{\tilde{h}}_{k}^{H}w_{j}\right|}^{2}+{\left\|F_{k}^{H}\Theta \right\|}^{2}\sigma^{2}+\sigma_{0}^{2}}\geq t$$

By introducing an explicit function of θ, P(6) can be rewritten as below:

(14a) $$P(7):\;\underset{\theta }{\max}\;t.$$

(14b) $$st.\;\theta^{H}H\theta +\theta^{H}D_{r}\theta \sigma^{2}\leq P_{R}\;\;\;\;\;\;\;\;\forall r\in R$$

(14c) $$\frac{(1-\xi_{k}){\left|b_{k,k}+\theta^{H}a_{k,k}\right|}^{2}}{\sum_{\begin{array}{c} j=1\\ j\neq k \end{array}}^{K}\left(1-\varsigma_{k}){\left|b_{k,j}+\theta^{H}a_{k,j}\right|}^{2}+\sum_{j=1}^{K}\xi_{k}{\left|{\overset{\sim }{h}}_{k}^{H}w_{j}\right|}^{2}+(\theta^{H}c_{k}c_{k}^{H}\theta \right)\sigma^{2}+\sigma_{0}^{2}}\geq t$$

where $\theta^{H}=\Theta 1_{RN}\in C^{1\times RN}$, $(\xi_{r}⊗I_{N}{)}^{H}F(\xi_{r}⊗I_{N})=H$, $\sum_{l=1}^{L}\sum_{j=1}^{K}[diag(G_{lr}w_{lj}){]}^{H}diag(G_{lr}w_{lj})\}=F$, $(\xi_{r}⊗I_{N}{)}^{H}(\xi_{r}⊗I_{N})=D_{r}$, $d_{k}^{H}w_{j}=b_{k,j}$, $diag(F_{k}^{H})Gw_{j}=a_{k,j}$, $diag(F_{k}^{H})=c_{k}$, $\xi_{r}\in R^{1\times R}$ represents the vector having 1 as its rth element and 0 for all remaining elements.

**Proof.**  Please see Appendix A. □

With the introduction of an auxiliary variable $\bar{\theta }$, P(7) is presented below:

(15a) $$P(8):\;\underset{\bar{\theta }}{\max}\;t$$

(15b) $$st.\;{\bar{\theta }}^{H}R_{\xi }\bar{\theta }\leq P_{R}$$

(15c) $$\begin{array}{c} (1-\xi_{k})[{\bar{\theta }}^{H}R_{k,k}\bar{\theta }+{\left|b_{k,k}\right|}^{2}]\geq \\ t[(1-\xi_{k})\sum_{j\neq k}^{K}({\bar{\theta }}^{H}R_{k,j}\bar{\theta }+{\left|b_{k,j}\right|}^{2})+\sum_{j=1}^{K}\xi_{k}{\left|{\tilde{h}}_{k}^{H}w_{j}\right|}^{2}+{\bar{\theta }}^{H}R_{c,c}\bar{\theta }] \end{array}$$

where $R_{k,j}=\left[\begin{array}{c} a_{k,j}a_{k,j}^{H},\;\;a_{k,j}b_{k,j}^{H}\\ b_{k,j}a_{k,j}^{H},\;\;\;0 \end{array}\right]$, $\bar{\theta }=\left[\begin{array}{c} \theta \\ 1 \end{array}\right]$, $R_{c,c}=\left[\begin{array}{c} c_{k}c_{k}^{H}\sigma^{2},\;\;\;\;\;0\\ 0,\;\;\;\;\;\;\;\;\;\;\;\;\sigma_{0}^{2} \end{array}\right]$, $R_{\xi }=\left[\begin{array}{c} H+\sigma^{2}D_{r},\;\;\;\;\;0\\ 0,\;\;\;\;\;\;\;\;\;\;\;\;\;\;0 \end{array}\right]$.

By applying the properties of the matrix trace operator, we obtain:

${\bar{\theta }}^{H}R_{k,j}\bar{\theta }=tr(R_{k,j}\Psi )$, ${\bar{\theta }}^{H}R_{\xi }\bar{\theta }=tr(R_{\xi }\Psi )$, ${\bar{\theta }}^{H}R_{c,c}\bar{\theta }=tr(R_{c,c}\Psi )$, where $\Psi =\bar{\theta }{\bar{\theta }}^{H}$.

We transform P(8) into its equivalent form given below:

(16a) $$P(9):\;\underset{\Psi }{\max}\;t$$

(16b) $$st.\;tr(R_{\xi }\Psi )\leq P_{R}$$

(16c) $$\begin{array}{c} (1-\xi_{k})[tr(R_{k,k}\Psi )+{\left|b_{k,k}\right|}^{2}]\geq \\ t[(1-\xi_{k})\sum_{j\neq k}^{K}(tr(R_{k,j}\Psi )+{\left|b_{k,j}\right|}^{2})+\sum_{j=1}^{K}\xi_{k}{\left|{\tilde{h}}_{k}^{H}w_{j}\right|}^{2}+tr(R_{c,c}\Psi )] \end{array}$$

(16d) Ψ≥0

(16e) rank(Ψ)=1

where the constraints (16b)–(16d) are convex. The constraint (16e) is nonconvex. Then we employ the SDR to relax (16e) [29] and reformulate P(9) as:

(17a) $$P(10):\;\underset{\Psi }{\max}\;t$$

(17b) $$st.\;tr(R_{\xi }\Psi )\leq P_{R}$$

(17c) $$\begin{array}{c} (1-\xi_{k})[tr(R_{k,k}\Psi )+{\left|b_{k,k}\right|}^{2}]\geq \\ t[(1-\xi_{k})\sum_{j\neq k}^{K}(tr(R_{k,j}\Psi )+{\left|b_{k,j}\right|}^{2})+\sum_{j=1}^{K}\xi_{k}{\left|{\tilde{h}}_{k}^{H}w_{j}\right|}^{2}+tr(R_{c,c}\Psi )] \end{array}$$

(17d) Ψ≥0

Obviously, P(10) is a conventional convex semidefinite program (SDP) that can be tackled with available optimization solvers. The bisection search method, employed for P(4), is also applicable and efficient for solving Problem P(10). The optimal solution Ψ to P(10) is not necessarily to be rank-one. Consequently, we employ Gaussian randomization to derive an approximate solution to the problem [21]. In Algorithm 2, we present the complete solution procedure.


**Algorithm 2**: Proposed Algorithm for Addressing P(10)**Input:** All channels $d_{l,k}^{H},G_{l,r},\;F_{r,k}^{H}$, the initial $W_{opt}$ and $\xi_{k}$. **Output:** RIS Reflecting Precoding $\Theta_{opt}$. 1: Initialize the minimum and maximum bounds for $\zeta_{\min}$ and $\zeta_{\max}$ for the variable in the objective function mentioned in (17a). Choose a tolerance δ=0.1. 2: Let $\varsigma =(\varsigma_{\min}+\varsigma_{\max})/2$. Tackle the SDP problem using the CVX solver:                             find: Ψ                                      s.t. $tr(R_{\xi }\Psi )\leq P_{R}$                                                                           (18a)                                            $\begin{array}{c} (1-\xi_{k})[tr(R_{k,k}\Psi )+{\left|b_{k,k}\right|}^{2}]\geq \\ t[(1-\xi_{k})\sum_{j\neq k}^{K}(tr(R_{k,j}\Psi )+{\left|b_{k,j}\right|}^{2})+\sum_{j=1}^{K}\xi_{k}{\left|{\tilde{h}}_{k}^{H}w_{j}\right|}^{2}+tr(R_{c,c}\Psi )] \end{array}$   (18b)                                            Ψ≥0                                                                                       (18c) 3: If the above problem is feasible, then let $\zeta_{\min}=\varsigma$, otherwise, set $\zeta_{\max}=\varsigma$. 4: if $\varsigma_{\max}-\varsigma_{\min}\leq \delta$, output Ψ. Otherwise, return to step 2. 5. Decompose matrix Ψ into its eigenvalues and eigenvectors, resulting in $\Psi =U\Sigma U^{H}$. Then, generate a set of 1000 candidate solutions $\overset{\sim }{\theta }=U\Sigma^{1/2}r$, where $r\sim CN(0,I_{RN+1})$ 6: Choose $\overset{\sim }{\theta }$ which maximizes the minimum of achievable rate                         $\frac{\left(1-\xi_{k})[{\bar{\theta }}^{H}R_{k,k}\bar{\theta }+{\left|b_{k,k}\right|}^{2}\right]}{(1-\xi_{k})\sum_{j\neq k}^{K}({\bar{\theta }}^{H}R_{k,j}\bar{\theta }+{\left|b_{k,j}\right|}^{2})+\sum_{j=1}^{K}\xi_{k}{\left|{\overset{\sim }{h}}_{k}^{H}w_{j}\right|}^{2}+{\bar{\theta }}^{H}R_{c,c}\bar{\theta }}$ ∀k∈K             (19) from 1000 suboptimal solutions. 7. Stop and output the optimized reflection coefficient matrix $\Theta_{opt}$ of the rth RIS.


According to [26], the complexity for Algorithm 2 is $O(Iter_{SDP}(p^{3}q^{3.5}+p^{2}q^{2.5}+p^{3}q^{0.5}))$, where p=K+1, q=RN+1 and $Iter_{SDP}$ represent the iteration count required for Algorithm 2 to converge.

## 4. Simulation Results

Simulation results are provided to evaluate the effectiveness of the proposed algorithm in the following. 

We investigated a system with K=4 users served by L=4 APs, where all APs, RISs and users were deployed within the $100\times 100\;m^{2}$ square area. The four APs were positioned at (100 m, 0 m), (−100 m, 0 m), (0 m, 100 m), and (0 m, −100 m) respectively. Four RISs were randomly placed within a circular area of 50 m radius. Users were also uniformly distributed inside the same region. The heights of APs, RISs and users were set as 15 m, 30 m and 1.65 m, respectively. We used the classical three-slope model as in [30] to model the large-scale fading coefficient, with noise power of $\sigma^{2}=-120\;\mathrm{dBm}$.

**Figure 2:**
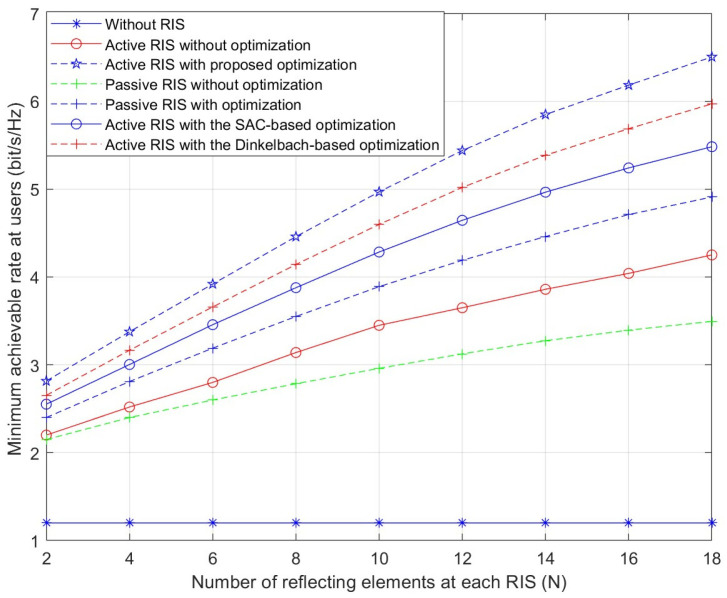
Minimum achievable rate versus the number of reflecting elements.

Figure 2 shows the correspondence between the max-min achievable rate and RIS element count. The simulation data first demonstrate the max-min achievable rate increases with N. In comparison to active RIS scheme without optimization, the presented scheme of active RIS with optimization can significantly improve the rate performance. Furthermore, one can observe that as N increases, performance gaps between the two scenarios mentioned above gradually widen. Increasing N from 6 to 18 results in an approximately 14% greater performance gain from the proposed algorithm. The proposed scheme demonstrates clear superiority over all benchmark methods. It is also noteworthy that the proposed scheme consistently outperforms two other algorithms for RIS precoding design—the Dinkelbach-based algorithm [21] and the SAC-based algorithm [18]—across the entire range of N. This clear performance margin further validates the superiority of the proposed optimization approach over existing benchmark methods.

**Figure 3:**
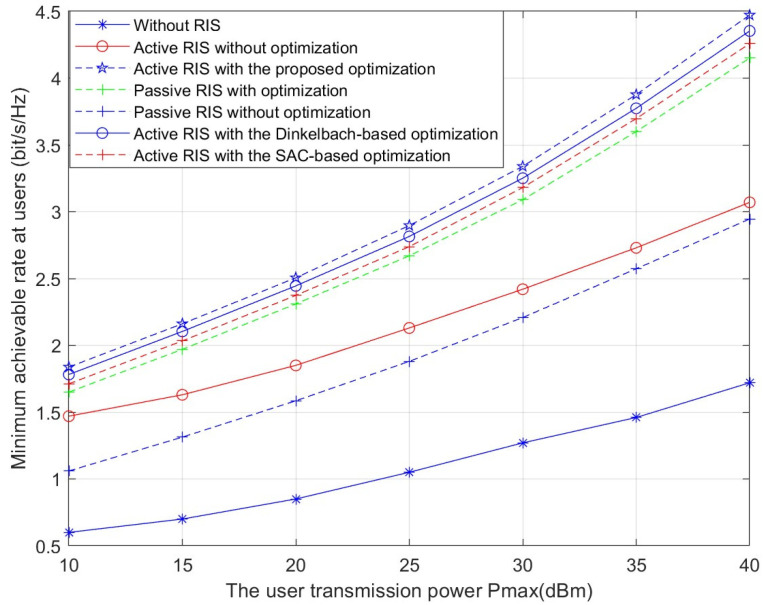
Minimum achievable rate versus the user transmission power Pmax.

Figure 3 presents the minimum achievable rate versus the maximum transmit power $P_{l}$. With higher maximum transmit power, the minimum achievable rate rises for both active and passive RIS-aided systems. Across all values of $P_{l}$, the proposed scheme demonstrates a consistent and substantial advantage over all benchmark methods. Compared to the scheme with active RIS without optimization, the proposed optimization algorithm can improve the rate performance by approximately 38%. Under identical conditions, active RIS demonstrates superior performance over the passive RIS. This improvement stems from the active RIS amplifying the signal to further decrease the double-fading effect. This demonstrates that we can use active RIS instead of passive RIS in CF-mMIMO systems to attain a higher minimum rate under the same conditions. Furthermore, when compared to two other optimization methods—the Dinkelbach-based algorithm and the SAC-based algorithm for RIS precoding design—the proposed approach still maintains a clear performance edge.

**Figure 4:**
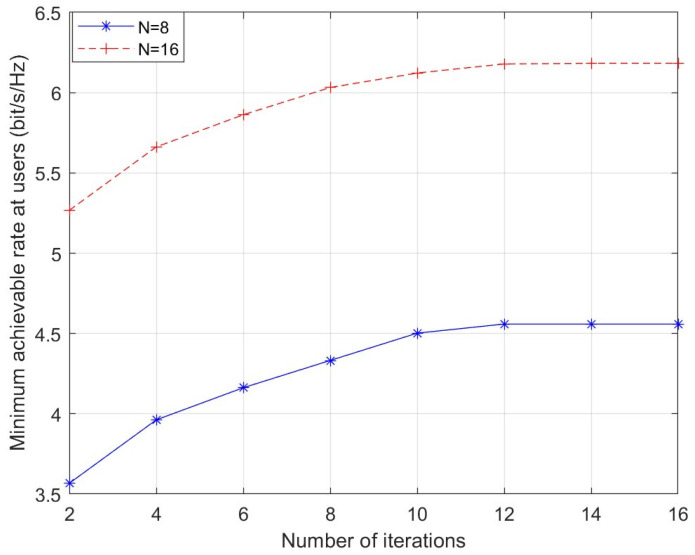
Convergence behavior of the proposed algorithm.

Figure 4 presents the proposed scheme’s convergence behavior. The minimum achievable rate exhibits a steady rise along with the increasing number of iterations, and then gradually slows down until it converges.

**Figure 5:**
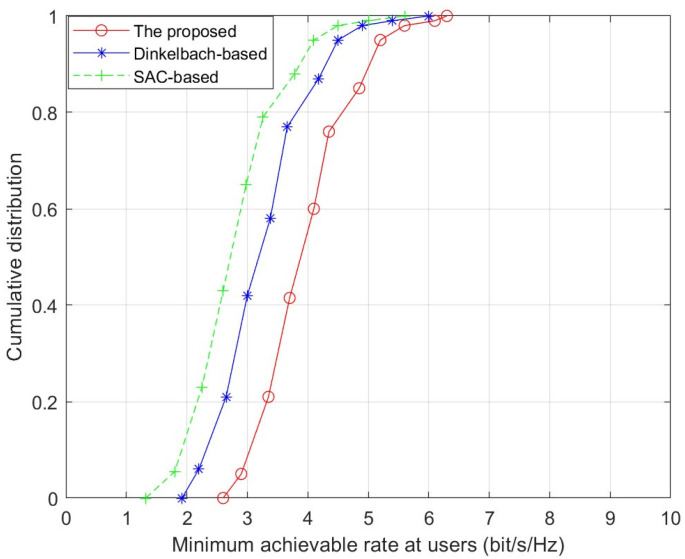
Cumulative distribution of the minimum achievable rate.

Figure 5 shows the cumulative distribution function (CDF) of the minimum achievable rate for three RIS precoding design algorithms, given N = 8 elements. In the three algorithms, the 95%-likely minimum achievable rates are 2.9 bit/s/Hz, 2.2 bit/s/Hz, 1.82 bit/s/Hz, respectively. The proposed algorithm consistently delivers superior performance compared to Dinkelbach-based algorithm and SAC-based algorithm. 

Table 1 presents the average CPU runtime versus the number of reflecting elements. As shown in Table 1, with the increase in N, the average CPU runtime increases for the three algorithms. This is because the computational complexities of all schemes rise as N increases. Moreover, it can be observed that the proposed scheme incurs higher CPU runtime than the other two schemes, consistent with the complexity analysis provided in the previous section. Dinkelbach-based algorithm has moderate complexity, with a complexity of $O(I_{DS}KR^{3}N^{3})$ where $I_{DS}$ is the number of Dinkelbach iterations [8]. SAC-based algorithm has very low complexity during inference, with a complexity of $O(HRNK+H^{2})$ [31], where H denotes the hidden layer width of the neural networks, but its training phase is computationally intensive. However, it only needs to be trained once to quickly provide solutions.

## 5. Conclusions

This paper investigates a downlink CF-mMIMO network assisted by an active RIS with imperfect CSI. We formulated a max-min achievable rate optimization problem and constructed a two-step optimization algorithm by leveraging SDP and SOCP, which includes optimizing AP transmit beamforming and RIS reflecting precoding. The introduced optimization algorithm significantly surpasses existing benchmarks with respect to the minimum achievable rate for active RIS-aided CF-mMIMO networks, as validated by simulations.

## Figures and Tables

**Table 1:** Average CPU runtime (seconds).

	*N* = 4	*N* = 8	*N* = 12	*N* = 16
The proposed	8.653	9.673	11.363	14.256
Dinkelbach-based	6.631	7.753	9.261	11.562
SAC-based (inference)	0.0036	0.0065	0.0081	0.0126

## Data Availability

The data presented in this study are available upon request from the corresponding author.

## Abbreviations

The following abbreviations are used in this manuscript:

6G	Sixth Generation
AP	Access point
RIS	Reconfigurable intelligent surface
WSR	Weighted sum-rate
SDP	Semidefinite program
SOCP	Second-Order Cone Programming

## Appendix A

(1) Compute $h_{k}^{H}w_{j}$:
  by defining $\theta^{H}=\Theta 1_{RN}\in C^{1\times RN}$, $d_{k}^{H}=[d_{1k}^{H},d_{2k}^{H},\cdots d_{Lk}^{H}]$, and $G=[G_{1},G_{2}\cdots G_{L}]$
  then:

$$\begin{array}{cc} h_{k}^{H}w_{j} & =\sum_{l=1}^{L}[d_{lk}^{H}+\theta^{H}diag(F_{k}^{H})G_{l}]w_{l,j}\\ & =d_{k}^{H}w_{j}+\theta^{H}diag(F_{k}^{H})Gw_{j}\\ & =b_{k,j}+\theta^{H}a_{k,j} \end{array}$$

where $d_{k}^{H}w_{j}=b_{k,j}$, $diag(F_{k}^{H})Gw_{j}=a_{k,j}$

(2) Compute ${\left\|F_{k}^{H}\Theta \right\|}^{2}$:
  by defining $diag(F_{k}^{H})=c_{k}$
  $$\begin{array}{cc} {\left\|F_{k}^{H}\Theta \right\|}^{2} & ={\left\|\theta^{H}diag(F_{k}^{H})\right\|}^{2}\\ & =\theta^{H}c_{k}c_{k}^{H}\theta \\ & =tr(\theta^{H}c_{k}c_{k}^{H}\theta ) \end{array}$$
(3) Compute $\sum_{l=1}^{L}\sum_{j=1}^{K}{\left\|\Theta_{r}G_{lr}w_{lj}\right\|}^{2}$:
  by defining $\sum_{l=1}^{L}\sum_{j=1}^{K}[diag(G_{lr}w_{lj}){]}^{H}diag(G_{lr}w_{lj})\}=F$
  $$(\xi_{r}⊗I_{N}{)}^{H}F(\xi_{r}⊗I_{N})=H,\;\theta_{r}^{H}=\Theta_{r}1_{N}\in C^{1\times N}$$
  then:

$$\begin{array}{cc} \sum_{l=1}^{L}\sum_{j=1}^{K}{\left\|\Theta_{r}G_{lr}w_{lj}\right\|}^{2} & =\sum_{l=1}^{L}\sum_{j=1}^{K}\left\|diag(G_{lr}w_{lj})\theta_{r}\right\|\\ & =\theta_{r}^{H}\{\sum_{l=1}^{L}\sum_{j=1}^{K}[diag(G_{lr}w_{lj}){]}^{H}diag(G_{lr}w_{lj})\}\theta_{r} \end{array}$$

$$\sum_{l=1}^{L}\sum_{j=1}^{K}{\left\|\Theta_{r}G_{lr}w_{lj}\right\|}^{2}=\theta^{H}(\xi_{r}⊗I_{N}{)}^{H}F(\xi_{r}⊗I_{N})\theta$$

where $\theta_{r}=(\xi_{r}⊗I_{N})\theta$, $\sum_{l=1}^{L}\sum_{j=1}^{K}{\left\|\Theta_{r}G_{lr}w_{lj}\right\|}^{2}=\theta^{H}H\theta$

(4) Compute ${\left\|\Theta_{r}\right\|}^{2}\sigma^{2}$:

$$\begin{array}{cc} {\left\|\Theta_{r}\right\|}^{2}\sigma^{2} & =\theta_{r}^{H}\theta \sigma^{2}\\ & =\theta^{H}(\xi_{r}⊗I_{N}{)}^{H}(\xi_{r}⊗I_{N})\theta \sigma^{2}\\ & =\theta^{H}D_{r}\theta \sigma^{2} \end{array}$$

where $(\xi_{r}⊗I_{N}{)}^{H}(\xi_{r}⊗I_{N})=D_{r}$, $\theta_{r}^{H}=\Theta_{r}1_{N}\in C^{1\times N}$.
